# Environmental Policy Integration in the Energy Sector of China: The Roles of the Institutional Context

**DOI:** 10.3390/ijerph17249388

**Published:** 2020-12-15

**Authors:** Xiang Ruan, Rong Sheng, Tuo Lin

**Affiliations:** 1 Department of Political Science, East China Normal University, Shanghai 200062, China; mruan@shnu.edu.cn; 2 Shanghai Institute of Global Cities, Shanghai Normal University, Shanghai 201234, China; 3 Institute of Eco-Chongming, East China Normal University, Shanghai 200062, China; 4 Institute of Developmental and Strategic Studies, East China Normal University, Shanghai 200062, China; xmlin1026@163.com; 5 School of Urban & Regional Science, East China Normal University, Shanghai 200062, China

**Keywords:** environmental policy integration, green energy in China, facilitating institutional context

## Abstract

The rapid growth of China’s renewable energy market and production capacity has attracted worldwide attention. Environmental policy integration in the energy sector and the institutional background behind this growth have seen little examination. In this paper, we present an assessment of environmental policy integration (EPI), attempting to reveal how the institutional factors facilitate EPI in the energy sector of China. A qualitative analytical framework involving normative, organizational, and procedural dimensions, incorporating multiple pieces of quantitative evidence, was applied. The results show that an ambitious and long-term normative vision covering political will, social backing, and cultural foundation in China is indispensable to the EPI process in the energy sector. The energy agency’s trans-sector cooperation in policy-making has been established to overcome the sectoral compartmentalization. China’s EPI in energy has a relatively complete and stable regulating system but, at the same time, it is expected to obtain more benefits from market cultivation and public participation. In this process, advantages such as the co-evolution of the green energy innovation, market, and society do exist; however, this market-oriented approach may bring the risk of economic and societal disturbances when interest-driven production capacity growth far surpasses market and societal requirements. This potential risk needs to be handled and prevented by strong governmental guidance and support. The continuous ambitious and long-term visioning of EPI, sufficient governmental funds, and a proactive industrial plan for renewable energy, are suggested.

## 1. Introduction

Due to increasing environmental and climate pressure, sustainable development has become a critically important and common goal across the world, especially in developing countries such as China. To achieve sustainable development, various social and economic sectors are required to undergo sustainable transitions. Policy integration is argued to be critical to this transition [1,2]. Environmental policy integration (EPI) is one important field of policy integration that deals with the integration of environmental concerns in socioeconomic policy targets. EPI is defined as the integration of environmental objectives into various sectors such as energy and agriculture, among others; however, the current status of EPI is believed to be less satisfactory in practice [3,4]. Some scholars proposed ways to enhance EPI performance by highlighting the roles of the government [5] and the participatory efforts of the public [6]; however, it is worth noting that EPI is primarily implemented at the institutional level, even though it is often initiation by policy. The institutional context here refers to the broad contextual background and factors with the potential to influence policy formulation and implementation. Though not always embodied in detailed policies, the institutional context potentially has an impact on the real-world effect of policy. This is especially true of policy integration because trade-offs are inevitable in the process of combining the needed policy objectives. More research is needed to contribute to the institutional perspective and identify the institutional roles in EPI.

This paper attempts to explore the institutional roles and conditions facilitating EPI, specifically in the energy sector of China. The energy sector, as a fundamental source for human survival and development, has also been involved in the aforementioned transition, aiming to be greener, cleaner, and more renewable. A variety of countries have implemented EPI strategies in energy sectors [2] and this has aroused many academic debates [6,7,8]. The previously examined cases of EPI in energy sectors are mostly related to European countries, despite the geography of energy consumption shifting towards Asia. Two decades ago, Europe and North America occupied 40% of the total global energy demand, which will be completely reversed by 2040 [9]. Thus, the sustainable transition of the energy sector in Asian countries is of great concern worldwide. China is among the leading countries vigorously integrating environmental concerns into the energy sector. It is among the world leaders in solar photovoltaic (solar PV), wind electricity, and electronic cars. During the 2019 to 2024 period, China’s renewable capacity expansion will be 40% of the global total and is expected to have almost half of all global distributed photovoltaic growth [10]. The study of China’s EPI in the energy sector is expected to contribute to this field by answering the following research questions: How do the institutional factors facilitate China’s EPI in the energy sector? Are there any problems hindering this process and what are the possible directions for tackling them, particularly in the renewable energy domain? Our research aim is to help improve the sustainable development of the Chinese energy sector and help the institutions in their implementation of EPI.

## 2. EPI: Policy and Institutional Perspectives

EPI refers to the consideration of environmental issues in other non-environmental sectors [11,12,13]. To achieve sustainable development, simple environmental protections and natural resource reservations are insufficient. The most important factor is the sustainable development of socioeconomic factors. The integration of environmental concerns into various sectors has been highlighted as a specific field of EPI. The energy sector is one of the fields in which EPI is most frequently discussed [6,7,8]. In the early period of its academic discussion, the “principled priority” is often considered to be environmental concerns due to sector growth [14]. This means that environmental interest is prioritized over other economic objectives rather than just adjusting or balancing conflicting policies. This assertion of environmental priority, though widely endorsed, proves to be difficult in actual EPI practice, and unfortunately, the prioritization of coordination in EPI may not always be achieved. Some later empirical studies show that EPI has a rather limited and indirect effect [15,16].

This has led researchers to re-examine and assess actual EPI processes, especially in individual countries. The existing research tends to follow the policy cycle and stage to examine the policies. The strategy, instruments, and outcomes within the policy cycle were evaluated with indicators for inclusion, coherence, weighting and reporting in an empirical study of Finnish EPI of technology policies [17]. Another study divided the policy cycle into policy input, processing, and output, and evaluated it with indicators for comprehensiveness, aggregation, and consistency in each period [18]. This same study argued that an ideal policy integration mode was to be built to see where the actual EPI stood in Japan and Norway, and how distant it was from the ideal standard. The policy stage was further extended and partitioned as involving the agenda setting, design, adoption, implementation, and evaluation in a study of EPI in the agricultural sector of Nigeria [13]. Furthermore, the indicators were also diversified. In addition to frequently used indicators for policy inclusion, coherence, and weighting, policy operationalization and capacity were considered in examining how biodiversity was integrated and prioritized in the non-environmental sectors of Peru [16]. All the above studies can be categorized into one strand of EPI assessment, which focuses more on the policy itself.

For another strand of EPI assessment, institutional conditions tend to hold more weight. Lenschow and Zito [19] built a framework covering organizational, procedural, and normative structures. Their operational evaluation criteria are too general to follow and some detailed points seem to overlap. Persson’s [20] research could be seen as an adaptation to the former framework and proposed three categories of factors to achieve EPI: normative, organizational, and procedural. The normative aspect includes high-level political commitment, societal backing, change of policy paradigms and time perspectives, etc.; the organizational aspect involves governmental structural change, coordination mechanisms, interaction with external actors, etc.; the procedural aspect concerns the implementation measures and changes of ordinary procedures. The evaluating criteria are clearly articulated and match well with the contemporary EPI barriers that are most likely to occur. The European Environment Agency (EEA) [21] proposed their own evaluation framework containing eight aspects, those being political commitment, organizational change, capacity building, tools to improve decision-making, implementation instruments, monitoring and other information support, sector policy greening, as well as changes of actual performance. The EEA’s [21] main points can be integrated to correspond with Persson’s [20] policy framework in 2004. The former is a more detailed expression of the latter framework. The EEA’s [21] framework, in particular, tried to identify EPI outcomes, whereas Persson’s [20] framework did not explicitly include this point. In the following studies, more institutional factors were explored. The leadership, cultural change, and capacity building were argued to influence EPI in Australia [22]. This study contributed a factor of cultural change, which may potentially influence the EPI process. Monitoring mechanisms and inter-sectoral policy coordination were proposed as the relevant institutional conditions for Swedish bioenergy development [23]. The EPI evolved under the shifting governance models and its correlation with a broad institutional background was highlighted [24]. The institutional perspective of EPI discusses the detailed conditions in the actual implementation of the policy portfolios, thus relating more to the real policy effect particularly in the long run as revealed in the abovementioned studies.

The above policy and the institutional aspects have no absolute segregation line. The policy measures are bound to be considered along with the context they are embedded in. Likewise, the institutional background has to be explored by centering around some certain policy measures; they are unified complexities. The difference between the two strands of the literature lies in their respective foci, whether probing into either policy more or the institutional conditions more.

Though researchers have made continuous efforts to improve EPI from an institutional perspective, the roles of the institutional context in formulating and implementing EPI need further exploration, especially in cases of countries with different political and economic backgrounds beyond the European context. In this paper, we present an assessment of EPI in the energy sector of China with a focus on the role of its institutional background, attempting to contribute to the aforementioned improvement. In this study, the institutional perspective was taken because it is more constructive in advancing the EPI process. It does not just center on the policy itself, but also extends to the pre-step of political will in EPI and the following substantial implementation as well as its real-world effects.

## 3. Institutional Analytical Framework and Methods

### 3.1. Building the Institutional Analytical Framework

In this paper, the evaluating framework that we used has three dimensions: normative, organizational, and procedural. We simplified and adapted Pearson’s [20] framework from 2004 to make it more compatible for operationalization.

The normative dimension refers to the general conceptualization of EPI in values and traditions [20]. High-level political will is a commonly agreed criterion that helps gain political momentum [25]. Furthermore, the stable or enduring expression of political will is believed to be important [26]. Social support is contended to be a pertinent research topic for implementing national sustainable strategies [27]. A new criterion that was newly added to the original framework is the cultural foundation, which might be inherited from traditions based on the relative abundance or lack of nature and resources. A country or region that is endowed with fewer natural resources is more likely to accept EPI and feels obliged to do so. The last two points constitute significant potential power to prompt the EPI process.

The organizational dimension exists to combat the sectoral compartmentalization in the governmental system [20]. It may involve governmental restructuring to bring substantial changes to the governmental system. The coordination between the various sectors and within one certain sector is also required in the administrative system [14]. The cooperation and communication mechanism/organizations are expected to provide inter-sector cohesion both horizontally and vertically.

The procedural dimension mainly covers EPI policy instruments in its implementation [20]. The policy instruments include regulatory, market-oriented, participatory, and information instruments [28,29]. Tech-based innovation and diffusion are also widely recognized as a critical component by policy-makers, researchers, and practitioners in economic growth and its contemporary sustainable transition. Here, the innovative factor is highlighted because technology appears to be a vital element that may co-evolve with institutional and social transformations [30]. In terms of the technology sector, the practical application of EPI must be beneficial to people and their lives. The concrete assessing framework used in examining China’s EPI in the energy sector is shown in Table 1.

### 3.2. Methodology

In our research, we conducted a case study based on a qualitative framework, incorporating quantitative indicators where necessary. Both qualitative and quantitative evidence are valuable in aiding EPI policy making and implementation processes. The data-based evidence is expected to be contextualized and interpreted in the institutional background. A policy and institutional oriented approach that involves qualitative material sorting, observation, and field visits as well as quantitative data was applied to obtain the information about the institutional roles in EPI of the case study.

The material was sorted based on multiple material sources, including quantitative and qualitative evidence such as the legal documents, policy documents, planning documents, press-release speeches and information provided by central governmental leaders, various miscellaneous reports, and the official websites of the ministries or authorities. One guiding principle for the selection of materials was that the materials can reliably and clearly demonstrate the facts, events, and attitudes that embody EPI and exert substantial or potential influences on the green energy transition in China. The observation and field visits of the Chongming eco-island in Shanghai, which has made lots of endeavors in the renewable energy field, aided the understanding and explaining of multiple materials and data. The quantitative data of the population and economy were retrieved from the open data of the World Bank. The quantitative data of the energy supply, consumption, electricity generation, and sustainable energy use were sourced from the International Energy Agency.

Based on the aforementioned framework, using our analytical approach, we examined three dimensions and six points. Firstly, the normative dimension covers content about the enduring high-level political commitment, social backing, and cultural foundation. Secondly, the organizational dimension includes governmental restructuring as well as the interdepartmental coordination and communication mechanisms/organization. Thirdly, the procedural dimension includes content about policy (regulatory, participatory, market, and information) as well a tech-based innovation growth and diffusion. For each of the six points, we examined the status of EPI in China with evidence from multiple sources.

## 4. Institutional Conditions Facilitating EPI in Energy Sectors in China

### 4.1. Overview and Some Facts of the Energy Sectors in China

Table 2 provides some basic details of China’s population, economy, and energy sector. Data for the Organization for Economic Co-operation and Development (OECD) countries and the world are also provided as comparative references. The population and economic data—as necessary background information—differ between countries, which directly contributes to the huge difference in the energy supply and energy consumption totals; however, some energy-relevant indicator values by population or by Gross Domestic Product (GDP) bear more comparativeness. The Total Primary Energy Supply (TPES) by GDP value of China is higher than those of the global average and OECD levels. The TPES per capita in China is slightly higher than the global average but much lower than the OECD countries. The same comparative pattern applies to electricity consumption per capita among these countries. China’s electricity consumption per capita is higher than the global average but much lower than the OECD level.

**Figure 1:**
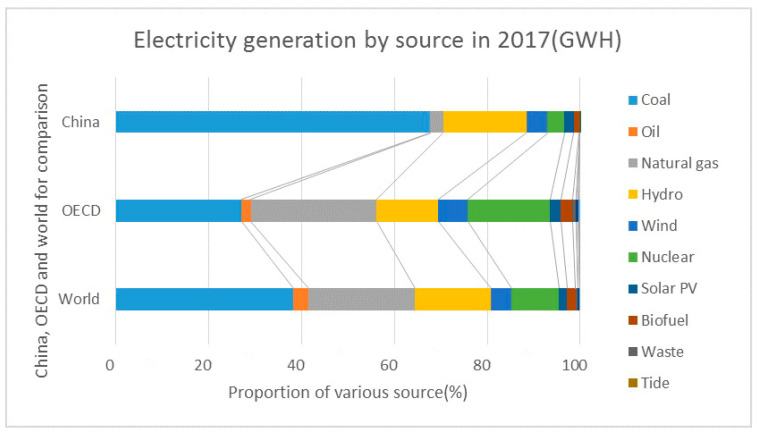
Electricity generation proportion in China by source in 2017 compared with the Organization for Economic Co-operation and Development (OECD) and world. Note: Relevant data are from country profiles of the International Energy Agency (IEA) [32]. In the above figure GWH is shortened for gigawatt hours and Solar PV for solar photovoltaic.

Fossil fuels still constitute major sources of electricity generation in China when compared with the world and OECD countries (see Figure 1). The total share of coal and oil used for electricity generation in China accounts for nearly 70%, whereas for OECD countries is only just over 20%, and for the world, over 40%. In terms of a sustainable transition to renewable energy, China is behind in terms of the amount of renewable energy in the total energy consumption compared with the global average; however, the amount of solar PV and wind electricity generation from 2010 to 2017 in China grew at a very fast pace, much higher than the global and OECD levels. The solar PV electricity generation in China has grown 185.9 times from 2010 to 2017—for comparison, OECD countries grew 7.54 times and for the world average growth is 12.8 times. Wind electricity generation in China has grown 5.6 times from 2010 to 2017—OECD countries only grew 1.59 times and the world average has grown 2.3 times. These data indicate China’s progress and great potential in renewable energy development. This country possesses some obvious comparative advantages in the world green energy transition and has carried out effective EPI measures situated within a favorable institutional background. To determine the facilitating factors or hinderances for the transition to sustainable development, we applied our analytical framework to an institutional analysis.

### 4.2. Examining Institutional Roles to Foster EPI

#### 4.2.1. Political and Public Visioning

China is a country with vast quantities of land and diversified resources, despite the relatively low per capita possession. Given the huge energy demand from rapid development, China is motivated to undergo a substantial development and energy transition. In the last decade, the Chinese government proposed achieving a balanced integration of economic, political, cultural, social, and ecological development. The Constitution of the Communist Party of China (Amendment) passed at the 18th National Congress of the Communist Party of China added the construction of ecological civilization to the Party Constitution. This suggests that the political will of integrating the environmental and ecological concerns will act as an enduring political goal. A review of the previous 12 Five-Year Plans shows the central government’s ever-growing attention to green energy development [33]. The current 13th Five-Year Plan proposed the construction of a modern energy system, focusing on reducing energy consumption and increasing energy efficiency. It is a manifestation of EPI being greatly and stably valued in a high-level political commitment.

**Figure 2:**
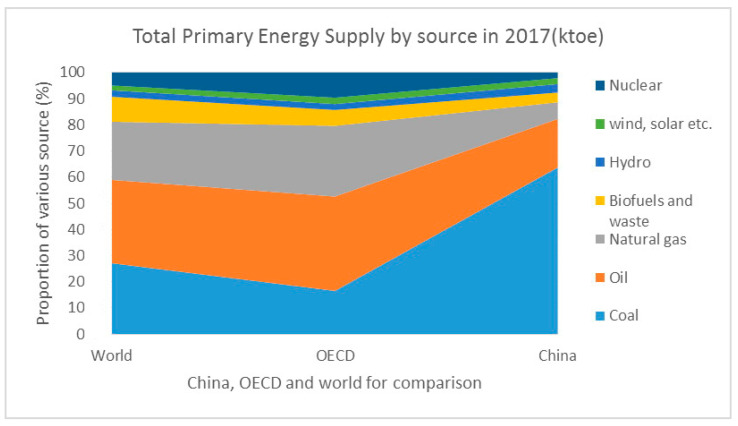
Total Primary Energy Supply by source in 2017. Note: Relevant data are from country profiles of the International Energy Agency (IEA) [32].

Coal and oil have been the basic source of China’s energy system for a long time. Now, China is undergoing a transition from an energy system based on fossil fuels to one based on renewable and nuclear energy sources [27]. According to the World Bank, China’s energy-savings were 57% of the global total from 1990 to 2010. Although coal and oil still counted for over 80% of the Total Primary Energy Supply (TPES), green energies such as biofuels, wind, and solar have gained stable places in the energy structure (as seen in Figure 2). For the majority of coal and oil, China has formulated rigid standards for its consumption total and density. Natural gas is widely used as an alternative to coal, especially in regions with higher concentrations of economic activity and population such as the Yangtze River Delta in East China. Though some researchers argue that the energy-intensive economic structure is hard to alter in the short term [27], China still vigorously promotes the transition of the energy system to a sustainable one, especially in terms of its orientation towards the goal of low greenhouse gas emissions.

The public in China has a growing awareness of energy saving and the green energy transition. The previous extensive energy development and consumption paradigm is undergoing a substantial change. In rural China, rural residents are undergoing a transition, turning to renewable energy when choosing their water heaters [34]. In urban areas, new energy-efficient transportation modes are widely promoted in people’s daily lives. In the past ten years, 4.5 million new energy-efficient motorcars have been promoted and used, accounting for over 50% across the world [35]. Electric and hybrid motorcars are regarded both as a new economic booster and a method to relieve environmental pressures, especially in metropolises like Shanghai and Beijing. The cultural traditions of Chinese people have a long history of respecting and interacting with nature. They rely on natural resources to make their livelihood. It is particularly so in some ecologically fragile and environmentally sensitive areas impacted by rapid urbanization and industrialization in recent decades. Recent empirical research found that cultural background, environmental consciousness, and educational background had an influence on a consumer’s willingness to pay for energy-efficient and energy-saving household appliances [36]. An increasing number of people realize this problem, which is beneficial for a sustainable energy transition.

#### 4.2.2. Inter-Departmental Coordination

In China, the National Energy Administration (NEA) is authorized to manage the energy sector as a governmental agency. The National Energy Administrative is supervised by the National Development and Reform Commission (NDRC). In terms of the administrative structure, the NEA is at a level lower than that of Ministry or Commission. In 1988, the Energy Ministry was established, but later canceled, in the 1990s. In 2008, the NEA was established and was responsible for energy-related affairs. Back in 1988, the electricity supervision functions were designated to the newly established Energy Ministry; however, in 2003, an independent National Electricity Supervision Commission (NESC) was established. Ten years later, in 2013, the duties of the NESC were combined with those of the NEA. Thus, in terms of governmental functional integration, the energy agency in China focuses more on the energy sector itself and the relevant electricity generation. It does not cover other major resource or infrastructure issues.

This does not mean that the energy sector in China lacks coordination in administrative structure. The NDRC, which the NEA is led by, plays an important role in connecting and coordinating the relevant organizations, especially in the fields of developmental and market-oriented activities. For example, in 2019, the NDRC led to issue a policy of Promoting the Bio Natural Gas Industrialization [37]. This policy initiative requires trans-sectoral efforts for the joint promotion of the industrialization of a new energy source. Agencies that were included in this promotion were the NEA, the Ministry of Finance, the Ministry of Natural Resources, the Ministry of Ecology and Environment, the Ministry of Housing and Urban–Rural Development, to name just a few. Furthermore, in 2019, the NDRC and NEA jointly initiated the promotion of the Power Consumption Guarantee Mechanism for renewable energies [38]. As the economy continues to slow, the consumption demand for electricity decreases; however, the production capacity of renewable energies is ever increasing. Electricity generated by renewable energy might be undermined when challenged by increasing competition among various energy supplies. Therefore the NDRC and NEA required provinces to prioritize consuming renewable energy. These two examples illustrate how the NDRC acts as a critical bridge to help the NEA cooperate with other ministries or provinces.

The above can be regarded as indirect coordination; there are also direct coordination links between the NEA and other governmental agencies in environmental policymaking. In 2010, the Ministry of Environmental Protection issued a policy of Promoting Joint Governance of Air Pollution and Improving the Regional Air Quality [39] with several ministries and agencies, including the NEA. It is led by the Ministry of Environmental Protection and the NEA is part of the joint issuers. In this sense, whether directly cooperating with other governmental departments or indirectly guided by the higher-level ministry, a joint policymaking mechanism has been established to address sectoral compartmentalization. Inter-departmental coordination provides a platform on which multiple governmental agencies can cooperate in promoting EPI in the energy sector.

#### 4.2.3. Policy Portfolio

China’s regulation of the energy system has been increasingly entrenched by legislative and policy instruments of green energy promotion rather than rudimentary legislation of fossil resources [40]. Currently, basic energy law in China has not been formally issued and its draft was released to seek public comments in April, 2020. Furthermore, in the energy sector, China has a relevant series of energy legislation including the Electricity Law (amended in 2015) [41], the Energy Conservation Law (amended in 2016) [42], the Coal Law (amended in 2013) [43], the Mineral Resources Law (amended in 2009) [44], and the Renewable Energy Law (amended in 2009) [45]. Regarding other regulatory policies, the most representative at the national level is the Five-Year Plans for National Economic and Social Development. In the 13th Five-Year Plan [46], the national government laid out bans and standards, strictly controlling the amount and density of energy development and consumption. The total energy consumption is required to be within five billion tons of standard coal. The energy-saving standard system is requested to cover all major industries and equipment. As for clean and renewable energy, the government regulates to implement clean energy alternative projects for small and medium-sized coal-fired facilities, urban villages, and urban–rural areas. Traditional energy such as coal is required to be limited and progressively replaced by natural gas in different regions. Coal is required to be used in a cleaner and more effective way. Other renewable energies such as solar, wind, and biomass are favored and supported.

The participatory instruments target two groups. One is to encourage the public to undertake the National Energy Conservation Action Plan [47], comprehensive energy cascade utilization, and distributed energy supply system. The other is for third-party operation entities to provide technical consultation, systematic design, equipment making, project implementation, and operation services, alongside energy contracts management, in the green energy domain. Market-oriented and information measures are indispensable, though the regulatory and participatory policy instruments are more visible in the green energy sector. The government specifies, in the Renewable Energy Law (amended in 2009) [45], that the full indemnificatory acquisition of renewable energy electricity is applied, which is a powerful guarantee for the sustained growth of the renewable energy market. In the 13th Five-Year Plan [46], the government plans to establish an initial allocation system of energy-use with a paid-use mechanism and to cultivate the trading market. Regarding cleaner energy, the government plans to free the prices within the competitive link of natural gas and implement progressive residential natural gas pricing. More social resources are planned to be introduced into the competitive domain of natural gas usage. The government proposed to build an online energy consumption monitoring system.

Energy innovation technology is proved to be crucial in China’s transition to a sustainable energy system in empirical studies [48,49]. Strengthening the Research and Development (R&D) of radically innovative green energy technologies is highlighted in various countries [50,51]. Because of China’s rapid development, renewable energy technology in China still has much room to improve. In the 2017 Global Cleantech Innovation index results, China ranked in 18th place, which was a slight upward move from 19th place in the 2014 list [52]. This might be a comparative indicator revealing the relatively weak innovation ability of China in general. The government, in its 13th Five-Year Plan [46], highlights the technological upgrading and transformation of boiler (kiln), lighting, and motor systems. It planned to undertake new technologies to support the green transformation of coal electricity. It also mentioned accelerating the development of new nuclear equipment and small nuclear power systems. Energy-saving tech is expected to go through research, demonstration, and promotion in order to fully achieve its economic and social effect; therefore, the industrialization of new technology and equipment, such as waste heat power generation, small gas turbines, and other energy-saving technologies is highly valued in the innovation system.

## 5. Co-Evolution of Green Energy Innovation, Market, and Society and the Potential Risk

It has to be acknowledged that China has formulated laws governing renewables, and laid rigid standards for traditional energy use; however, its focal point lies in encouraging the public to use greener energy and involving more market players in the development of renewable energy. The Renewable Energy Law (amended in 2009) [45] states that the country encourages the entities and individuals to install and use solar water-heating systems, solar warming and cooling systems, and solar PV electricity generation systems; however, under the State-led model [53], the central government intended to mobilize more public and market resources in this transition. Public resources are expected to be included in green energy development and, at the same time, the public could be cultivated to build a green society. The same applies to the market elements. More enterprises are involved in the green energy industry and more benefits are to be obtained through a market-oriented green transition. This is supposed to be a co-evolution. A third factor contributing to this process is green energy tech innovation. The diffusion and transfer of EPI tech innovation in China are difficult because its ecological industries are less developed. EPI technology and innovation in the energy sector in China lack good demonstration, promotion, and transference. The level of income is found to act as a turning point at which the green energy technology can be truly converted to green productivity [54]; therefore, tech diffusion is a highly localized matter because tech application is embedded in social, economic, and cultural conditions; thus, a publicly involved and market-oriented green energy development is beneficial to innovation and promotion. A comparative example might be Israel, which possesses extensive innovation competence and potential. In the green energy sector, promising and applicable technologies include the coal-to-gas fuel switch for electricity generation, solar PV, and solar thermal and energy efficiency in buildings. The current green energy sector transition in Israel is mostly driven by the supply side of the technological configuration [55]; however, application and diffusion are not enough. Most citizens have not acquired a sense of living with new levels of technology around them. The huge social potential has not yet been realized [56]. The concerns of Israel are centered on the opportunities for the implementation of most technologies, given various local natural and social conditions [57].

What is different in China is that this country has been attempting to cultivate tech innovation, market, and society as part of the same process. This, of course, is attributed to China’s advantage of owning a big domestic market. It constitutes a co-evolution of green energy innovation, the green energy market, and a green energy society. The green energy transition is not only about rigid control but realizing more market vitality as well. The public is able to experience more benefits from new energy technology and is willing to input more resources.

However, a potential risk does exist behind the prosperity and growth under the market pull. In the interest-driven renewables capacity expansion process, the overheated market is confronted with the downsizing of demand, following the slowing of economic growth [58]. The regulation system is challenged by increasingly uncertain conflicts of interest arising from various new energies and even fossil fuel sources in the competition for market share. If this tension cannot be relieved, it could negatively affect the energy economy and social stability. In light of policies seeking to tackle this issue, the good momentum of renewable energy development is expected to be maintained for a long period; however, the growth of renewable energy and its production capacity needs to be adjusted to strike a balance with dynamic consumption needs as well as other traditional energy supplies. While the energy system transitions to environmentally friendly solutions, the levels of coal burning are expected to steadily increase in recent years [59]; therefore, governments will face complex difficulties in adjusting and guiding this painful process. In order to confront this dilemma, several policy suggestions are made as follows. Above all, governments should continuously build on the aforementioned ambitious and long-term envisioning of EPI. Confidence in renewable energy in particular needs to be further consolidated. Furthermore, sufficient funds from governments are needed to support renewable energy consumption in this process. Once the production capacity of renewables fails to achieve the expected economic gains, the renewable energy field will likely downscale and experience market instability; therefore, governmental funds are helpful to further support the innovation and development of relevant technologies. Furthermore, a proactive industrial plan in renewable energy should be established and revised according to the dynamic evolution of renewables and its relation to traditional energy.

## 6. Conclusions

China’s renewable energy market has experienced rapid growth, which could be counted as the outstanding achievement of China’s green energy transition. The Solar PV electricity generation in China has grown 185.9 times and wind electricity generation in China has grown 5.6 times from 2010 to 2017 as compared with much lower figures in OECD countries and the world level. Examining the institutional context in which EPI is embedded is expected to provide new insights for better EPI in the energy sector in China.

Based on a qualitative analytical framework incorporating multiple pieces of quantitative evidence, China’s EPI in energy sector was assessed from the institutional perspective. The results show how the institutional factors facilitate EPI in the energy sector in China. Great efforts and progress have been made. Normatively, the EPI process is activated by high-level political commitment, which is capable of mobilizing the resources needed. As for the social backing and cultural foundation, China has a tradition of respecting nature and saving habits—this is an advantage that can be harnessed to widely advance a greener energy system. Organizationally, a joint policymaking mechanism, involving multiple governmental agencies acting as policy co-issuers, has been established in its green energy transition. Inter-departmental coordination is beneficial in deciding the EPI tradeoffs and combat the sectoral compartmentalization. Procedurally, China has formulated a variety of measures to put EPI into effect. The regulating system, centering on legislation and major national policies, remains the anchor of national EPI ideology. Based on a relatively complete regulating system for EPI, China tries to obtain more benefits from market cultivation and public participation. It constitutes a co-evolution of green energy tech innovation, the green energy market, and a green energy society; however, a potential risk exists when production capacity grows much faster than the market and societal requirements. Strong governmental guidance and support are needed to tackle this problem. Suggestions may involve the continuous ambitious and long-term visioning of EPI, sufficient governmental funds, and a proactive industrial plan for renewable energy.

Further studies might include further analysis of the co-evolution process among tech, market, and society in energy EPI in China and other countries in the world. More exploration can be made in terms of tech-based innovation and its relevant market and social changes as well as their mutual interactions. This process is vital to gain more market efficiency and social welfare in the sustainable energy transition.

## Figures and Tables

**Table 1:** Institutional framework adjusted to assess environmental policy integration (EPI) in China’s green energy field.

Dimensions	Operationalizing Criteria
Normative	Enduring high-level political commitment
	Social backing and cultural foundation
Organizational	Governmental restructuring
	Coordination and communication mechanisms/organization
Procedural	Policy instrument, e.g., regulatory, participatory, market and information
	Tech-based innovation breeding and diffusion

**Table 2:**
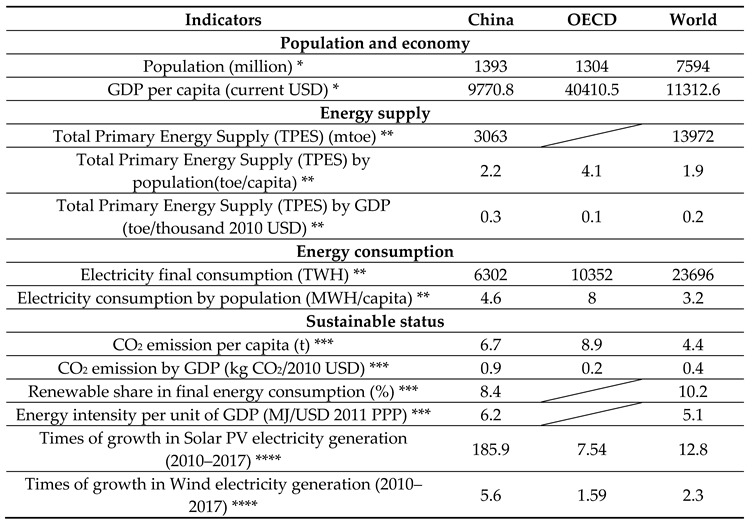
Some critical data of population, economy, energy supply, consumption, and sustainable conditions in China (for 2016–2018).

Notes: * Relevant data in 2018 were sourced from the open data of the World bank [31]. ** Relevant data in 2017 were sourced from the country profiles of the International Energy Agency (IEA) [32]. *** CO_2_ relevant data in 2017 and other relevant data in 2016 were sourced from the country profiles of the International Energy Agency (IEA) [32]. **** Relevant data in 2017 were sourced from the country profiles of the International Energy Agency (IEA) [32]. In the above table OECD is shortened for Organization for Economic Co-operation and Development, mtoe for million tons of oil equivalent, GDP for Gross Domestic Product and Solar PV for solar photovoltaic.
